# Antimicrobial Usage by Small-Scale Commercial Poultry Farmers in Mid-Western District of Masindi Uganda: Patterns, Public Health Implications, and Antimicrobial Resistance of *E. coli*

**DOI:** 10.1155/2023/6644271

**Published:** 2023-04-20

**Authors:** Majalija Samuel, Tony Fredrick Wabwire, Gabriel Tumwine, Peter Waiswa

**Affiliations:** College of Veterinary Medicine Animal Resources and Biosecurity, Makerere University, P.O Box 7062, Kampala, Uganda

## Abstract

**Background:**

Poultry production in Uganda is growing at a fast rate due to increasing demand, notwithstanding, poor husbandry practices, and diseases, prompting farmers to rear healthy productive flocks with antimicrobials. The study evaluated the knowledge and practices as regards the use of antibiotics among poultry farmers in Masindi district and determined the antibiotic susceptibility profiles of *E. coli* strains from chickens.

**Methods:**

A cross-sectional study using a closed-end questionnaire was conducted in 140 selected small-scale commercial poultry farms in Masindi district between June and December, 2020. Analyzed qualitative data were presented as frequencies, percentages, and their 95% confidence intervals (95% CI). Fecal swabs from chickens were inoculated onto a MacConkey agar, and *E. coli* was confirmed by standard biochemical tests. Antimicrobial susceptibility was determined by the disk diffusion method for 7 antibiotics.

**Results:**

Most farmers (74%) used antibiotics, mainly tetracycline (51.4%) and sulfonamides (28.6%), given to the chicks (45%), for both curative and prophylaxis purposes (80%), and via drinking water (67%). Farmers mainly used antibiotics recommended by the veterinarian (76.4%), more than relying on experience (10.7%), while 45% were involved in self-medicating the birds. On choosing the correct dosage, 45.7% read the instruction, and 42.9% consulted a veterinarian. Only 10.7% observed the drug withdrawal period, while 53.6% consumed eggs at home or sold eggs (35.7%) from birds under treatment. Of the 200 *E. coli* strains, 90 (45.0%) were resistant to one drug, 74 (37.0%) to two, and multidrug resistance to three classes of antibiotics was 36 (18.0%). Overall, *E. coli* resistance to tetracycline was (69.0%), ampicillin (37.0%), sulfonamides (36.0%), and to kanamycin (1.5%).

**Conclusions:**

The small-scale poultry farmers frequently use antimicrobial drugs, mainly tetracycline and sulfonamides for curative and prophylaxis. Thus, enforcing measures against antibiotic use supported by a strong veterinary service sector and farmers' training on judicious use of antimicrobials are needed.

## 1. Background

Almost all rural and peri-urban homesteads in Uganda are engaged in agriculture, a sector that employs over 70% of the population [1]. Around 35 million birds are reared, mainly as free-ranging indigenous chickens (80%), with the rest under the intensive management of exotic breeds [1]. In comparison to other livestock, poultry production is growing at a faster rate due to the high national and regional demands. Consumption of poultry and poultry products is acceptable in most cultures, supporting food security, nutrition, and livelihoods for the rural poor [2]. Regardless, poultry production faces severe challenges such as poor husbandry practices, low-quality feeds, sanitary and housing deficiencies [3, 4] associated with the emergence of pathogens, and high disease burden. Inevitably, farmers opt to use antimicrobials for treatment and/or prevention of diseases and promotion of rapid growth [5, 6].

Without doubt, the irrational use of antimicrobials in animals is a major contributor to increased antimicrobial resistance (AMR) [7, 8] and the emergence of multidrug-resistant (MDR) bacteria which is of global health concern [8, 9]. AMR hampers the treatment of life-threatening infections in both humans and animals. Imprudent use of antimicrobials can selectively promote the emergence of drug-resistant commensal bacteria in the gut [10, 11], which are excreted in feces, may contribute in food contamination and food-borne infections in humans [12–14].

Even though poor countries in sub-Saharan Africa (SSA) contribute a significant proportion to the global antibiotic use [15–17], the lack of reliable data on antimicrobial use in animals is an obstacle to the designing of mitigation strategies. Empirical data show the gravity of the problem; for instance, arbitrary determination of antimicrobial dosage before administration was reported by 93% of poultry keepers in Nigeria [18]. Similarly, the use of antibiotics without prescription and failure to observe antibiotic withdrawal periods were also reported [19, 20]. Recent reports in Uganda indicate that 97% and 66% of farmers frequently used antibiotics in their poultry farms from Wakiso [21, 22] and Nakaseke [23] districts, respectively, which are gotten from drug shops without prescriptions from veterinarians [19, 20]. In general, the abuse of antimicrobial usage is exacerbated by weak enforcement of policies in Uganda [24, 25].

Effective handling of AMR requires a multisectoral collaboration of human, animal, and environmental sector players, defined as one health [26, 27]. At the national level, the Uganda government has established a multisectoral one health coordination office as a coordination frame and a National Action Plan to guide and counter AMR [28]. This is also in tandem with global efforts through the Quadripartite collaboration on AMR, consisting of the Food and Agriculture Organization (FAO), the World Organization for Animal Health (OIE), the World Health Organization (WHO), and the United Nations Environment Programme (UNEP) aims at antimicrobials stewardship [29–31]. The WHO Global Action Plan on AMR [29] and the European Medicines Agency (EMA)/Antimicrobial Advice Ad Hoc Expert Group (AMEG) [32] explicitly aim to strengthen knowledge and evidence-based practices through surveillance and research to guide the development of appropriate strategies that ensure antimicrobial stewardship in human and animal health [33].

Clarifying the gaps in knowledge and practices of poultry farmers as regards antimicrobial use patterns is needed prior to designing meaningful mitigation strategies. The study evaluated the knowledge and practices as regards the use of antibiotics among poultry farmers in Masindi district and determined the antibiotic susceptibility profiles of *E. coli.* The identified knowledge and skill gaps of the farmers will be used to create awareness and influence prudent behavioral change on antimicrobial use.

## 2. Methods

### 2.1. Design and Setting of the Study

**Figure 1:**
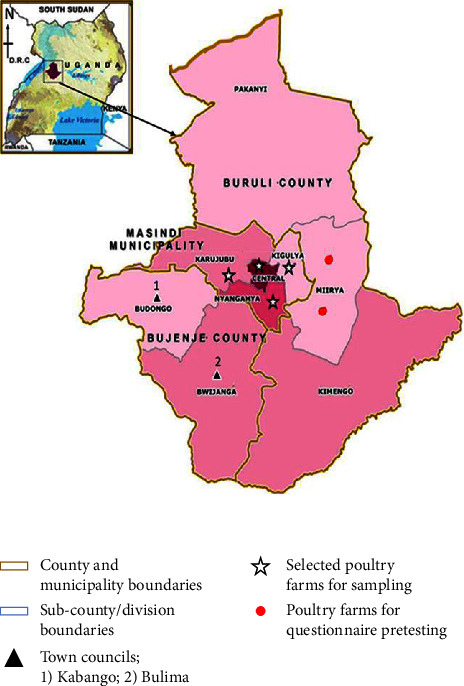
Map of Masindi district showing Masindi municipality (study area), Bujenje and Buruli counties. Adopted from Uganda Bureau of Statistics [34].

The study was conducted in Masindi district, located between coordinates 1° 41“ 0″ N and 31° 44“ 0″ E in Mid-Western Uganda, 214 kilometers from Kampala, the capital city of Uganda. The district covers an area of 7,443 sq. km, with 6332 sq. km of arable land. It has a favorable climate with a bimodal rainfall pattern and an annual long-term average rainfall of 1,304 mm. The district is divided into 5 subcounties of Budongo, Bwijanga, Kimengo, Miirya, and Pakanyi and two urban town councils of Bulima and Kabago, in Bwijanga and Budongo subcounties, respectively. In addition, the urbanised municipality comprises 4 divisions: central (urban) and three peri-urban divisions of Karujubu, Kigulya, and Nyangahya (Figure 1). A total population of 292,951 people is majorly rural (68%) and engages in agriculture as the main economic activity (https://www.masindi.go.ug/district/population). Most households keep local chickens on a free-range system, with an intensive deep liter system for exotic birds, especially the layers confined to the urban and peri-urban areas. As one of the districts in Uganda's Albertine oil region, commercial poultry keeping is a target enterprise to meet the growing demand for eggs and meat. Small-scale commercial poultry farmers keep between 100 and 1000 exotic birds in an intensive management system, which also provides additional income for the urban and peri-urban dwellers in the district.

A cross-sectional study using a closed-end questionnaire survey was conducted in selected small-scale commercial poultry farms in Masindi between June and December, 2020. Prior to the survey, the study team met with local veterinary officials and leaders of poultry farmers to discuss the survey plans. The study population was small-scale commercial poultry farmers raising layer birds under a deep litter intensive husbandry system. A total of four divisions/subcounties were selected from Masindi district (central division, Kigulya, Karujubu, and Nyangahya) within the urban and peri-urban areas based on ease of access and the large number of farmers that reared exotic layer poultry.

The sample size was calculated at a 95% confidence level with the assumed prevalence of antibiotic use of 10% and margin of error at 5.0% of all farms, which gave 139 farms. From the selected divisions/subcounty, 140 households'/poultry farms were selected from a list of farms (*n* = 400) obtained from Masindi district veterinary office (this was estimated based on their experience as there are no official records on the number of farms). The first farm was randomly selected, while the second and subsequent farms were selected by consecutive sampling until the required number was obtained. A farm was included if the owner or an adult responsible for taking care of the layer birds was present and agreed to provide verbal informed consent. Farms were excluded if owners or a responsible adult could not be located at the time of the visit; however, the next available farm was considered for the interview if it met the selection criteria.

Prior to administering the questionnaire, the purpose of the study was outlined. The team procured sachets and bottles of antibiotics available from the local veterinary drug shops in the area as samples which were displayed before the farmers to guide in the identification of familiar drugs used with their farms. Oral consent to participate in the study was obtained, and all consenting farmers present on the day of the interview participated. A closed-ended questionnaire was administered by a research assistant conversant in Runyoro, the local language used in the area. The focus of the questionnaire was on poultry farmers' knowledge and practices believed to be associated with the imprudent use of antimicrobials. The questionnaire aimed to explore participants' socio‐demographic characteristics, common diseases experienced at the farm, the person(s) responsible for treating sick birds, and drugs commonly used among others. The questionnaire was pretested on 10 individuals in Miirya subcounty with a similar setting in the district. Direct observations and farm records were also used to gather additional information related to antimicrobial usage practices.

### 2.2. Bacterial Culture and Antibiotic Susceptibility Testing

Two composite fecal swabs obtained from five fresh chicken droppings in each poultry farm were placed in sterile tubes containing Stuart transport media and transported on ice in a cooler box to the Microbiology Laboratory in the College of Veterinary Medicine, Animal Resources and Biosecurity, Makerere University, Kampala. Samples were inoculated onto a MacConkey agar (MAC) (Remel, Lenexa, KS, USA) and incubated aerobically at 37°C for 18−24 hrs. Presumptive *E. coli* was confirmed by standard biochemical testing using API20E (Analytical Profile Index; BioM6rieux, Hazelwood, MO).

Antimicrobial susceptibility of *E. coli* on Mueller-Hinton agar was determined by the disk diffusion method [35] for seven antibiotics: tetracycline (30 *μ*g), neomycin (10 *μ*g), gentamicin (10 *μ*g), kanamycin (30 *μ*g), ampicillin (10 *μ*g), trimethoprim (5 *μ*g), and sulfonamides (200 *μ*g) (Oxoid TM). To determine antimicrobial susceptibility of bacterial isolates, the mean zones of inhibition of three replicates were measured in millimeters and interpreted as susceptible (S), intermediate (I), or resistant (R) according to CLSI [35]. Isolates showed resistance to at least three classes of antimicrobials were categorized as multidrug resistant (MDR) [36]. *E. coli* ATCC 25922 was used as a control strain.

### 2.3. Data Management and Analysis

The collected data were coded and entered into an Excel worksheet (Microsoft® Excel for Windows, 2017). Data cleaning and analysis were carried out in Statistical Package for Social Sciences (SPSS version 17) to obtain descriptive statistics for each variable. Results were presented in the form of frequencies, percentages, and their 95% confidence intervals (95% CI) to determine the extent of antimicrobial use in poultry production in the study area. Antibiotics used for poultry production in Masindi district were grouped into four categories A to D based on EMA/AMEG classification [32] (i) category A: avoid includes antibiotics that are currently not authorized for veterinary use and may only be given to companion animals under exceptional circumstances; (ii) category B: restrict, antibiotics are critically important in human medicine, and their use in animals should be restricted to mitigate the risk to public health, and only used when there are no antibiotics in categories C or D; (iii) category C: caution includes antibiotics for which alternatives in human medicine generally exist, but only few alternatives are available in certain veterinary indications. Should only be used when there are no antimicrobial substances in category D that would be clinically effective; and (iv) category D: prudence includes antibiotics which should be used as first line treatments, whenever possible, which should be used prudently and only when medically needed.

## 3. Results

Socio-demographic characteristics of the respondents A total of 140 poultry farmers involved in the study included 101 (72.1%; CI: 63.9–79.4) males and 39 (27.9%; CI: 20.6–36.1) females, with the majority 95 (67.9%; CI: 59.5–75.5) and 68 (48.6%; CI: 40.0–57.2) having secondary education and age group between 31 and 50 years, respectively, and 72% (CI: 63.9–79.4) with experience of more than 10 years (Table 1).

### 3.1. Antibiotic Use Practices in Poultry Production in Masindi District

A total of 103 (74%; CI: 65.5–80.7) poultry farmers used antibiotics with 37 (26%; CI: 19.3–34.5) reported to have not used antibiotics on their farm in the past two months. It was reported that a majority (48.6%; CI: 40.0–57.2) always used antibiotics while 57 (40.7%; CI: 32.5–49.3) rarely, and 8 (5.7%; CI: 2.5–11.0) sometimes use antibiotics; only 5.0% (CI: 2.0–10.0) have never used antibiotics on their farms (Table 2).

The antibiotics were frequently given to the chicks (45%; CI: 36.6–53.6), mainly for curative (40%; CI: 31.8–48.6) or prophylaxis purpose (40%; CI: 31.8–48.6) for growth promotion (20%; CI: 13.7–28.0) and via drinking water (67%; CI: 58.7–74.8) or feeds (33%; CI: 25.1–41.3). The personnel involved in the treatment of the sick birds were veterinarians and animal health workers (51.4%; CI: 42.8–60.0), farm owners (45%; CI: 35.9–52.9), and peer farmers (4.3%; CI: 1.6–9.1). Aside from antibiotics (55%; CI: 46.4–63.4) of the poultry farmers, other natural remedies are used including Aloe vera and green pepper to treat newcastle disease and coccidiosis.

### 3.2. Types of Antimicrobials Used in Poultry Production in Masindi District

Regarding the type of antibiotics frequently used in poultry production, tetracycline (51.4%; CI: 42.8–60.0) and sulfonamides (28.6%; CI: 21.3–36.6) were mostly used. These two classes of drugs were included in category D (prudence) based on AMEG classification. Additionally, neomycin (7.1%; CI: 3.5–12.7) and gentamycin (2.1%; CI: 0.04–6.1) in category C (caution) were less likely to be used (Table 3).

### 3.3. Antibiotic Use Practices and Associated Public Health Risks

As regards the public health risks associated with antibiotic use practices, farmers mostly selected antibiotics, recommended by the veterinarians (76.4%). While 10.7% of the farmers used previous experience, 7.2% chose only available drugs and only 2.1% relied on drugs that were advertised (Table 4).

Almost equal proportion 45.7% (CI: 37.3–54.5) and 42.9% (CI: 34.5–51.5) read instructions or consulted a veterinarian for the correct dose of antibiotics to use. Nonetheless, 9.3% (CI: 5.0–15.4) depended on guesswork to determine the correct dosage (Table 4). Regarding the withdrawal period, it was reported that most of the eggs from birds under antibiotic treatment were consumed at home (53.6%; CI: 45.0–62.0) or sold in the market (35.7%; CI: 27.8–44.3), with only 10.7% (CI: 6.1–17.1) discarded as recommended (Table 4).

While 51.4% (CI: 42.1–59.3) used antibiotics without referring to its expiry date, 26.4% (CI: 19.3–34.5) were not aware of the expiry date, and only 22.1% (CI: 15.6–29.9) read and followed the expiry dates as recommended (Table 4).

### 3.4. Health Challenges Reported by Poultry Farmers in Masindi District

**Figure 2:**
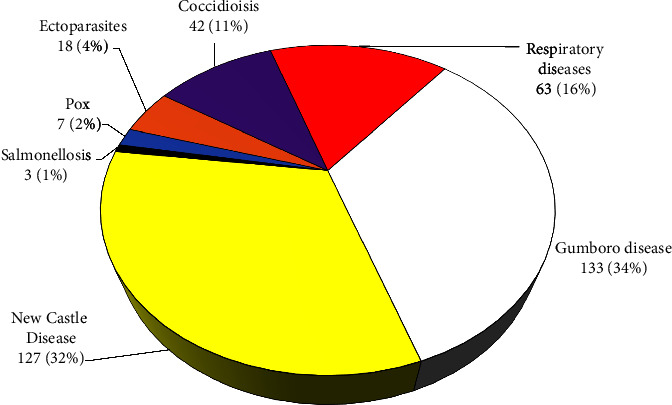
Common diseases and conditions on farms.

The flock size of small holder poultry farmers ranged between 100 and 300 birds, with a median of 240 birds. The three major health problems experienced on poultry farms were Gumboro (133; 34%), new castle disease (127; 32%), and respiratory diseases (16%). Coccidiosis and ectoparasites were also experienced by poultry farmers, among others (Figure 2).

### 3.5. AMR Profile of *E. coli*

Out of the 262 *E. coli* strains investigated, 200 (76.3%) were resistant to at least one drug with 138 (69%; CI: 62.3–75.0) being resistant to tetracycline, followed by ampicillin (37%; CI: 30.6–43.9) and sulfonamides (36%; CI: 30.6–43.9), respectively, and least to kanamycin (1.5%; CI: 0.51–4.32) as shown (Table 5).

**Figure 3:**
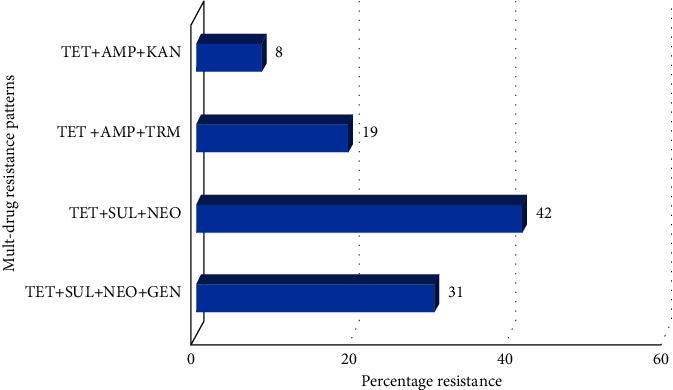
MDR patterns of *E. coli* from poultry in Masindi district.

Ninety (45%) of the 200 resistant isolates were only resistant to one drug; for TRI, TET, and AMP, the percentages were 18%, 31%, and 51%, respectively. While 62% were resistant to TET and SUL, 24% and 18% were resistant to TET and AMP or TRM, respectively. Overall, 36 (18%) of the bacterial isolates were resistant to at least one drug in three classes of antibiotics (multiple drug resistance). These include resistance to TET, SUL, and NEO (42%), TET, AMP, and TRM (19%) or KAN (8%). The remaining 31% of the isolates were resistant to TET, SUL, NEO, and GEN (Figure 3).

## 4. Discussion

The study provides crucial information on antibiotics use practices among small-scale commercial poultry farmers in Masindi district, which is useful in planning and implementation of strategies to mitigate the emerging AMR. As the demand of poultry products increases, farmers in Masindi district are motivated to produce more; however, this calls for practices that ensure rational use of antimicrobial drugs mindful of consumers' health concerns. A larger segment of the farmers (74%) use antibiotics and 48.6% always use antibiotics on their poultry farms. This was higher than previously reported in Tororo (15%) but comparable to Wakiso (74.0 versus 71.3%) in eastern and central Uganda [22], respectively. In an earlier study in Uganda, the antimicrobial use reported among livestock-keeping households was at 35% [37]. The contrasting findings of antimicrobial usage in our current study with the previous studies in Uganda could be due to knowledge differences among study populations. It also suggests an increasing trend in accessing and use of antimicrobials by farmers. Our findings are in agreement with reports of frequent antibiotic use among poultry farmers in Tanzania where 100% used antibiotics [38] and 65% in Abia State, Nigeria [39]. The shift in production practices from traditional [40] rearing of scavenging indigenous birds to commercial intensive exotic poultry production, which presumes that optimal health and production can be attained only with antimicrobials use, is the root cause of the problem. The need to find alternatives to antimicrobials or optimal practices that enhance health and productivity is what the future needs.

Arguably, the high frequency of antimicrobial usage could be attributed to the use of antimicrobials for curative and prophylaxis treatment, rather than for the promotion of growth. No growth promoters are used by poultry farmers in Masindi probably because they are not available in the market. Nevertheless, vitamin formulations containing tetracycline or sulfonamides are readily available for use. Typically, poultry reared under stressful conditions in overcrowded houses with inadequate ventilation and awful hygienic environments are highly predisposed to infection [41]. Most farmers opt to use antibiotics as a cover up for such poor animal husbandry practices, a justification which could be avoided. Farms that implement adequate biosecurity systems to limit undue distress in birds [42–44] can effectively maintain healthy productive flocks with minimal antimicrobial use. As well, our finding of excessive use of antimicrobials in poultry production raised public health concerns, which could lead to the transmission of resistant bacteria to humans and the environment [8, 9]. It is well documented that antibiotic-resistant pathogens shed from birds can be transmitted to humans and cause food-borne illness with severe sequelae [13].

The antibiotics frequently used in poultry were tetracycline and sulfonamides which were similar to the findings of earlier studies in Uganda [21, 22] and elsewhere in Tanzania [38], Nigeria [39], and Ghana [6]. In contrast, another report from poultry farmers, in Nigeria, indicated the frequent use of cotrimoxazole and neomycin in chicken production [45]. Although the drugs used might be different, category C and D antibiotics are readily available as cheap generics, accessible to farmers over-the-counter without prescription from veterinarians. In contrast, gentamycin, an antibiotic commonly available in an injectable formulation, is less likely to be used by poultry farmers.

Farmers treated sick birds without the involvement of veterinarians in 44.6% of the respondents and in most cases using antibiotics. Such practices are frequent in many countries in Africa [6, 21, 39] and could lead to under-dosing or over-dosing of birds. Often, farmers with more experience undertake treatment of their flocks and consult a veterinarian only after the treatment has failed, which would increase antibiotic misuse and the risk of drug resistance. This is in concurrence with a previous report from Ghana which reported that over 50% of poultry farmers relied on personal experience to administer antimicrobials to their animals [6, 46]. Probably, experienced farmers are biased towards seeking veterinary services in preference to consulting fellow farmers for advice in a bid to minimize the cost of treatment. This is because small-scale commercial poultry farmers may not be able to afford the services of veterinarians [47, 48] and opt for other alternatives. Overall, the enforcement of stringent measures and guidelines on antibiotic use under veterinary supervision is needed, notwithstanding the prevailing weak veterinary services in Uganda [24, 25, 49]. A section of farmers used herbal preparations to treat various poultry diseases, such as newcastle disease and coccidiosis. Herbal drugs are frequently used by poultry farmers to treat various illnesses in Uganda [50]. Despite the availability of vaccines against most of the prevalent viral diseases, a good portion of the farmers kept their birds on antibiotics most of the time. This irrational use of drugs is promoted by aggressive marketing of antibiotics, the ease of obtaining drugs without prescription, and administration without veterinary advice.

Most farmers read instructions or relied on the veterinarian's instructions to estimate the dosage of drugs. Since the majority had attained secondary education and were between 20 and 50 years, it can be surmised that they had a relatively good understanding of written instruction. Moreover, a majority of urban poultry farmers are working class with higher education background and can easily follow the instructions given by veterinarians. However, it was observed that farmers that frequently used veterinary advice were more likely to use higher frequencies of antibiotic-containing agents for treatment and prevention of infections [6]. This could be due to the fact that veterinarians are not readily available to supervise and oversee antibiotic use at the farm, which gives farmers the confidence to misuse the drugs left in their hands.

The observance of the antibiotic withdrawal period is required to ensure animal products are free from drug residues. Nonetheless, most poultry farmers in our study used antibiotics without considering the drug withdrawal period or the expiry dates, which was similar to another study in Ghana [6]. This in part shows a lack of adequate knowledge on the deleterious effects of consuming eggs with high concentrations of antibiotics but also suggested that farmers do not care about the public health implications of such a practice. Farmers deliberately ignore the withdrawal periods and sell prohibited eggs to limit financial losses [46]. A lack of monitoring from governmental agencies had been previously identified as a reason why withholding periods were not adhered to by farmers [51]. Largely, the limited facilities in Uganda cannot effectively test and monitor the presence of drug residues in eggs [25, 49].

Only a small proportion of the poultry farmers was aware of and followed the expiry date, suggesting the need for farmer sensitization. Furthermore, a majority of farmers were not acquiescent to observe the expiry dates of antibiotics. Use of expired drugs raises concerns of low efficacy and effectiveness which contributes to AMR. As earlier on, reported and expired oral pediatric antibiotics have been associated with high AMR in a study from Nigeria [47]. Thus, it is of necessity to have policies that ensure expired antibiotics are not administered in animals.

That 76.3% of *E. coli,* from the poultry in Masindi were resistant to at least one drug, was not surprising. This was comparable to an earlier study that reported 70% resistance in Uganda [52]. Other studies reported a higher prevalence of AMR of 88.4% [53] and 87% [54] of *E. coli* isolates in poultry in Uganda. Overall, 56% of the *E. coli* isolates were resistant to at least two antibiotics, suggesting that the exotic chicken from small commercial layers is beset by high level of AMR. The high AMR reported in this study is similar to previous studies reported in Uganda [53]. The emergence of drug-resistant bacteria in animal populations and their potential transfer to humans is of global public health concern [8, 9]. The likelihood of misuse is high in Uganda, a country with weak veterinary services and poor regulation of antibiotic usage [25, 49].

The commensal *E. coli* strains that colonize the gut of animals are used as indicator bacteria for changes within the microflora ecosystem [55]. The use of antimicrobials in poultry production can create selective pressure for *E. coli* leading to the emergence of antibiotic resistant strains in the gut [10, 11]. Thus, the observed AMR can be construed as a predictor of increased selective pressure as a result of excessive use of antimicrobial agents in poultry production. This can also be used as an early warning sign for the emergence of AMR in various species of microbes within the gut. This may require another study to investigate the susceptibility of various species of gut microflora to antibiotics.

Category C and D antibiotic drugs used in poultry production are simultaneously listed as drugs for treating various human illnesses in the country [56, 57]. There should be a clear justification with a reasonable need for their use in poultry health, as opposed to routine administration. Notably, prudent antimicrobial usage and curbing of the emerging resistance in livestock at the community and national scale would require interdisciplinary and multisectoral collaboration, referred to as one health, of different sectors: animal, human, and environmental health [27, 29–31]. Thus, through the National Action Plan (NAP), the government of Uganda has designed comprehensive strategies to contain and control AMR threats in the human and animal health sectors at the national level [28]. These efforts will in the long run prevent, slow down, and control the spread of resistant pathogens [28].

## 5. Conclusion

The small-scale poultry farmers in Masindi continue to use antimicrobial drugs, mainly tetracycline and sulfonamides for curative and prophylaxis treatments, without consulting veterinarians. The experienced farmers tended to avoid seeking veterinary services in a bid to minimize the cost of treatment. Adherence to the recommended drug withdraws period and expiry dates for drugs was glaringly lacking putting the consumers health at risk. Majorly, *E. coli* strains were resistant to multiple antimicrobial drugs suggesting increasing selective pressure due to excessive use of drugs in poultry production. The enforcement of stringent measures and guidelines on antibiotic use supported by a strong veterinary service sector in Uganda as well as farmers training on judicious use of antimicrobials are needed.

## Figures and Tables

**Table 1:** Characteristics of respondents.

Characteristics	Frequency/numbers	Percentage	95% CI
(a) Gender			
Males	101	72.1	63.9–79.4
Females	39	27.9	20.6–36.1
(b) Level of education			
None	11	7.9	4.0–13.6
Primary	33	23.6	16.8–31.5
Secondary	95	67.9	59.5–75.5
Tertiary	1	0.7	0.02–3.9
(c) Age group (years)			
>19	12	8.6	4.5–14.5
20–30	59	42.1	33.9–50.8
31–50	68	48.6	40.0–57.2
>50	1	0.7	0.02–3.9
(d) Experience in poultry farming (years)			
<5 years	4	2.9	0.78–7.2
5–10 years	35	25.0	18.1–33.0
>10 years	101	72.1	63.9–79.4

**Table 2:** Antibiotic use practices in poultry production.

Attribute	Frequency	Percentage	95% CI
(a) Have you given antibiotics to your birds in the past 2 months			
Yes	103	73.7	65.5–80.7
No	37	26.4	19.3–34.5
(b) How often do you use poultry antibiotics			
Always	68	48.6	40.0–57.2
Sometimes	08	5.7	2.5–11.0
Rarely	57	40.7	32.5–49.3
Never	07	5.0	2.0–10.0
(c) Stage of birds using antibiotics			
Chicks <2 month	63	45.0	36.6–53.6
Grower 2–6 months	40	28.6	21.3–36.8
Mature birds >6 months	37	26.4	19.3–34.5
(d) Reason for use of antibiotics			
Curative treatment of sick birds	56	40.0	31.8–48.6
Prophylaxis (preventing occurrence of sickness)	56	40.0	31.8–48.6
Promotion of growth	28	20.0	13.7–28.0
(e) Personnel involved in the treatment of sick birds			
Veterinarians and animal health workers	72	51.4	42.8–60.0
I treat first and consult a veterinarian after birds fail to recover	62	44.3	35.9–52.9
Consult fellow farmer	6	4.3	1.6–9.1
(f) How antibiotics administered to the birds?			
Via drinking water	94	67.0	58.7–74.8
Mixed in feeds	46	33.0	25.1–41.3
By injection	0	0	0.0–0.3
(g) Besides antibiotics, have you used local herbs to treat sick birds			
Yes	77	55	46.4–63.4
No	63	45	36.8–53.6

**Table 3:** AMEG categories of veterinary antibiotics used in poultry production in Masindi district.

AMEG category	Antimicrobial drug	Class	Frequency	Percentage	CI (95%)
D-prudence	Tetracycline hydrochloride	Tetracycline	72	51.4	42.8–60.0
D-prudence	Trimethoprim/Sulfadiazine, sulfamethoxazole, trimethoprim	Sulfonamide	40	28.6	21.3–36.8
D-prudence	Nitrofuran derivatives	Furazolidone	15	10.7	6.1–17.1
C-caution	Neomycin sulphate	Aminoglycoside	10	7.1	3.5–12.7
C-caution	Gentamicin sulphate	Aminoglycoside	3	2.1	0.04–6.1

EMA/AMEG classifies antibiotics used in animals into four categories A, avoid; B, restrict; C, caution; D, prudence. Category C (caution) is antibiotics for which alternatives in human medicine generally exist in the EU, but only a few alternatives are available in certain veterinary indications. These antibiotics should only be used when there are no antimicrobial substances in category D that would be clinically effective. Category D (prudence) includes antibiotics that are used for first line treatment with prudence and only when medically needed.

**Table 4:** Antibiotic use practices and associated public health risks.

Attribute	Frequency	Percentage	95% CI
(a) Criteria for selecting a drug and use			
Drug recommended by the veterinarian	107	76.4	68.5–83.2
Previous experience of getting good results	15	10.7	6.1–17.1
Only drug that is available	10	7.2	3.5–12.7
Advice from fellow farmer	5	3.6	1.2–8.1
Commonly advertised drugs	3	2.1	0.4–6.1
(b) Estimation of dosage during treatment			
Reading and following instructions	64	45.7	37.3–54.3
Rely on the veterinarian instructions	60	42.9	34.5–51.5
Depends on severity of the diseases	3	2.1	0.4–6.1
Guess the estimated dose	13	9.3	5.0–15.4
(c) Observance of withdrawal period			
Eggs of treated birds are sold	50	35.7	27.8–44.3
Eggs of treated birds are for home consumption	75	53.6	45.0–62.0
Eggs of treated birds are discarded or fed to dogs	15	10.7	6.1–17.1
(d) Observance of expiry date of antibiotics			
I read and follow the expiry date	31	22.1	15.6–29.9
I use the drug until it is used up without minding about the expiry date	72	51.4	42.8–60.0
I do not know that drugs can expire	37	26.4	19.3–345

**Table 5:** Overall AMR of *E. coli* isolates to selected drugs.

AMEG category	Antibiotic	Frequency (*n*)	Percentage (%)	95% CI
D	Tetracycline (TET)	138	69	62.3–75.0
D	Ampicillin (AMP)	74	37	30.6–43.9
D	Sulphonamides (SUL)	72	36	30.6–43.9
D	Trimethoprim (TRM)	33	17	12.0–22.3
C	Neomycin (NEO)	26	13	9.03–18.4
C	Gentamycin (GEN)	11	5.5	3.1–9.58
C	Kanamycin (KAN)	3	1.5	0.51–4.32

## Data Availability

The data supporting the current study are available from the corresponding author upon request.

## Abbreviations

AMEG: Antimicrobial advice ad hoc expert group
AMR: Antimicrobial/AMR
CI: Confidence interval
EMA: European medicines agency
AMP: Ampicillin
GEN: Gentamycin
KAN: Kanamycin
NEO: Neomycin
TRM: Trimethoprim
SUL: Sulphonamides
TET: Tetracycline.
